# Nurturing Healthy Smiles: Brazilian Immigrant Parents’ Perceptions and Parenting Practices of Healthy Eating to Promote Oral Health in Preschool-Aged Children: A Qualitative Study

**DOI:** 10.3390/children12070896

**Published:** 2025-07-07

**Authors:** Ana Cristina Lindsay, Maria Gabriela Miranda Fontenele, Adriana Bento, Steven A. Cohen, Mary L. Greaney, Denise Lima Nogueira

**Affiliations:** 1 Department of Urban Public Health, Robert and Donna Manning College of Nursing and Health Sciences, University of Massachusetts Boston, Boston, MA 02125, USA; 2 Faculty of Nursing, Federal University of Ceará, Fortaleza 60355-636, Brazil; maria.gabriela129@hotmail.com; 3 Brazilian Community Partner, University of Massachusetts Boston, Boston, MA 02125, USA; adrianabentousa@gmail.com; 4 Department of Public Health, College of Health Sciences, University of Rhode Island, Kingston, RI 02881, USA; steven_cohen@uri.edu (S.A.C.); mgreaney@uri.edu (M.L.G.); 5 Department of Nursing, Faculty Luciano Feijão, Sobral 62050-215, Brazil; deniseln2009@hotmail.com

**Keywords:** oral health, Brazilian immigrant parents, preschool children, diet, nutrition, qualitative methods

## Abstract

**Background/Objectives:** Oral health (OH) in early childhood is a key determinant of long-term well-being, shaped by parenting-related dietary and hygiene habits. While these influences are well-documented, they remain underexplored among Brazilian immigrant families in the United States (U.S.). Therefore, this study was designed to examine how Brazilian immigrant parents’ perceptions and practices regarding diet and oral hygiene affect their preschool-aged children’s OH. **Methods:** This qualitative study consisted of in-depth interviews with Brazilian immigrant parents of preschool-aged children (ages 2–5) living in the U.S. Individual, in-depth interviews were conducted via Zoom, recorded, and transcribed in Brazilian Portuguese. Two native Brazilian researchers experienced in qualitative methods conducted a thematic analysis of the transcripts in Brazilian Portuguese using MAXQDA, a qualitative data analysis software. The analysis focused on identifying key perceptions, parenting practices, and barriers related to children’s diet and OH. **Results:** Parents strongly associated sugary foods with poor OH, identifying sugar as a major contributor to dental issues. Both mothers and fathers reported limiting sugar intake and encouraging good oral hygiene practices. While parents prioritized educating their children on healthy habits, barriers such as time constraints, reliance on external childcare, and economic limitations affected the consistent implementation of strategies. **Conclusions:** Brazilian immigrant parents understand the importance of diet and oral hygiene in in supporting their children’s early OH but face barriers in broader social contexts, such as daycare, preschools, and communities. Interventions should aim to support parents in their caregiving roles while simultaneously addressing systemic and environmental obstacles. Public health efforts should account for cultural, economic, and contextual factors to more effectively support Brazilian immigrant families in promoting their children’s OH.

## 1. Introduction

Oral health (OH) in early childhood, a crucial determinant of long-term health outcomes, is significantly influenced by dietary and oral hygiene habits—both of which are shaped by parental knowledge and practices [1,2]. As children establish their eating and oral hygiene routines, parents play a central role in fostering their understanding of the importance of healthy eating and oral care and promoting these behaviors [3,4]. Although existing research highlights the significant connection between diet, oral hygiene, and OH, there remains a notable gap in understanding how parents, particularly those in immigrant communities, perceive and manage these relationships [4,5,6].

Immigrant populations, including Brazilians, are among the fastest-growing demographic groups in the United States (U.S.) [7,8]. Over recent decades, the number of Brazilian immigrants has steadily increased, with many settling in urban areas and contributing to the cultural and social diversity of local communities [7,8]. Despite their growing presence, Brazilian immigrants remain an understudied group, specifically in terms of health behaviors and OH practices. This research gap is particularly concerning, given the challenges Brazilian immigrant parents face in raising children in a new cultural and social environment while managing OH concerns such as early childhood caries and gum disease [9,10,11,12]. Furthermore, the lack of understanding of these practices has broader implications, as OH is closely linked to overall well-being [1,2,3,4,5].

Excessive sugar consumption has long been recognized for its negative effects on children’s OH, with sugary foods and drinks contributing to cavities and other dental issues [5,11]. While parents generally recognize the importance of limiting sugar intake and promoting oral hygiene, they often face challenges in consistently enforcing these practices [11,12]. Time constraints, busy family schedules, limited supervision outside the home, and economic barriers to accessing healthier foods are some of the obstacles parents encounter [13,14,15,16].

Although studies have explored the influence of parenting beliefs and practices on children’s OH across various populations, including U.S.-born families and some Latin American immigrants, there is a gap in research on how Brazilian immigrant families approach diet and oral hygiene, particularly in the context of acculturation [11,12,13,14,15,16]. Factors such as limited access to dental care, financial constraints, language barriers, and differences in dietary habits between Brazil and the U.S. may hinder parent’s ability to implement effective preventive OH measures for their children [13,14,15,16]. Additional external challenges—such as the widespread availability of sugary foods, limited supervision in schools and social settings, and the competing demands of work and family life—further hinder Brazilian immigrant parents’ efforts to establish consistent OH routines [9,10,15,16].

Brazilians represent one of the fastest-growing immigrant groups in the United States, with significant communities in states such as Florida, Massachusetts (MA), and New York [17]. Massachusetts is home to the second-largest Brazilian population in the United States, following Florida [18]. Unlike most Latin American immigrants, who speak Spanish, Brazilians are native Portuguese speakers—a linguistic distinction that can shape their experiences in navigating services, education, and community engagement. Like many other immigrant groups, Brazilians often face barriers in adapting to a new country, including challenges related to language access, employment, housing, healthcare, and legal status [17].

Given these complexities, it is crucial to understand how Brazilian immigrant parents perceive and manage the connection between diet and OH. Despite the critical importance of establishing good OH behaviors early in life, this group remains underexplored in current research. Therefore, this study aims to explore how Brazilian immigrant parents’ perceptions and parenting practices related to healthy eating and oral hygiene impact their preschool-aged children’s OH in the U.S.

## 2. Materials and Methods

### 2.1. Study Design

The present study is part of a larger mixed-methods project and builds on a previously published qualitative study using the same dataset and research team [19]; the current study focuses on a distinct research question and aspect of the data. This qualitative study employed in-depth, semi-structured interviews to explore perceptions and parenting practices related to how healthy eating and oral hygiene impact preschool-aged children’s OH [20]. Qualitative methods were used as they capture rich, detailed narratives and provide insights into the cultural and contextual factors influencing health behaviors, particularly in immigrant communities [21,22].

### 2.2. Participants Eligibility and Recruitment

Participants were purposively selected to ensure a range of experiences and perspectives among Brazilian immigrant families in Massachusetts (MA), a state with one of the largest Brazilian populations in the U.S. [9]. Inclusion criteria were (1) being a Brazilian immigrant mother or father, (2) being at least 21 years old, (3) having at least one child aged 2–5 years, (4) living in MA, and (5) residing in the U.S. for at least six months. Participants who did not meet the inclusion criteria were not eligible for the study. Additionally, families with children who had known atypical developmental delays (e.g., Autism Spectrum Disorder) were excluded, as parenting practices and oral health routines may differ in these contexts. The six-month minimum U.S. residency criterion was used to ensure participants had some exposure to the U.S. healthcare system and cultural environment. This residency threshold reflects the demographic characteristics of the Brazilian immigrant population in Massachusetts, which consists largely of new immigrants [18].

Potential participants were recruited through community organizations, social media groups (e.g., Facebook and WhatsApp groups), and snowball sampling, where participants referred others within their social networks to the study [23]. Interested participants called the number listed on the flyer to contact the research staff, who confirmed eligibility and scheduled interviews.

### 2.3. Data Collection

Prior to the start of each scheduled interview, which was conducted via Zoom, participants were informed about the study’s purpose, procedures, and their rights, including the right to withdraw at any time without penalty. Informed consent was obtained electronically before proceeding with the interviewer-administered questionnaire that assessed sociodemographic information (age, marital status, education, household income, and the number of children aged 2–5) as well as the Short Acculturation Scale for Hispanics (SASH) [24]. This 12-item scale measures language use, media consumption, and ethnic social relations, with higher scores indicating greater acculturation. A score of 2.99 or higher was considered indicative of higher acculturation levels [24].

Immediately after completing the questionnaire, participants participated in an in-depth qualitative interview, also via Zoom. This video conferencing platform allowed for face-to-face interaction while ensuring participant convenience and safety. Zoom enabled parents to join the interview from their preferred locations, minimizing barriers such as transportation and childcare.

To ensure rigor and credibility, the research team included members with expertise in qualitative research and a strong understanding of the participants’ cultural context [25]. The interviews were conducted in Brazilian Portuguese by native Brazilian researchers with experience in qualitative methods and working with immigrant populations. The interviewer also took field notes to capture non-verbal cues and contextual details during the interviews.

Three researchers (A.C.L., D.L.N. and M.G.M.F.) conducted the interviews, with the majority completed by the senior researcher (A.C.L.), a native Brazilian with extensive experience in qualitative interviewing. To ensure consistency across interviewers, the senior researcher (A.C.L.) led a structured training session prior to data collection, which included review of the interview guide, mock interviews, and group discussions on tone and probing techniques. Calibration procedures included periodic debriefings to review interviewing approaches and maintain consistency throughout the study.

Data collection was guided by a semi-structured interview guide [26], which was developed in Portuguese by experienced researchers who are native Brazilian Portuguese speakers with expertise in oral health, nutrition and feeding, and immigrant health. This process ensured cultural and linguistic appropriateness for the study population, Brazilian parents living in the U.S., and included careful attention to cultural relevance and language nuances to accurately capture participants’ experiences. The interview guide was pilot tested to refine questions and ensure clarity. Three participants (two mothers and one father) were involved in the pilot testing. Based on their feedback, minor revisions were made to improve flow and clarity, including reordering certain questions, rephrasing items for better understanding, and combining a few questions to reduce redundancy. These adjustments helped ensure that the final version of the guide was coherent, culturally appropriate, and easy for participants to follow.

The semi-structured interview guide, informed by the Social–Ecological Model (SEM) [27] and the Extended Health Belief Model (EHBM) [28,29] and the existing literature, included open-ended questions to explore and elicit detailed narratives about parents’ perceptions and practices related to their children’s OH, with a focus on parental oral hygiene and dietary practices for their children, as well as cultural influences on these behaviors [6,11,12,13,14,15,16,17,18].

The SEM provided a framework for examining how factors at multiple levels—individual (e.g., knowledge and attitudes), interpersonal (e.g., family influence), organizational (e.g., access to services), community (e.g., cultural norms), and policy—shape OH-related behaviors. The EHBM guided our exploration of individual-level motivators and barriers, focusing on constructs such as perceived susceptibility to OH issues, perceived severity of these issues, perceived benefits of preventive behaviors, perceived barriers to action, cues for action, and self-efficacy. These models were selected due to their complementary strengths: SEM captures the broader contextual and structural influences on behavior, while HBM allows for a deeper understanding of individual perceptions and motivations. This combined approach was particularly well-suited to exploring the multifaceted determinants of parental OH behaviors. For example, questions addressing parental beliefs about the consequences of poor oral hygiene were guided by the EHBM’s perceived severity and susceptibility constructs, while questions exploring family routines, community influences, and access to dental care reflected SEM’s multi-level framework.

### 2.4. Data Analysis

A professional transcription service transcribed the Zoom audio-recorded interviews verbatim in Brazilian Portuguese. The transcripts were reviewed and de-identified. A codebook was developed using the EHBM and SEM as a framework for coding and analysis [30,31]. Transcripts were systematically coded, with new codes added to the codebook as they emerged during analysis. The analysis was conducted using MAXQDA, a qualitative data analysis software that supported systematic coding, organization, and text retrieval [32]. The initial coding was primarily conducted by one bilingual researcher (D.L.N.) and reviewed by a second (A.C.L.), with discrepancies discussed and resolved through consensus to ensure accuracy and consistency in the coding process [20,30,31].

The analysis followed an iterative process, with the research team reviewing codes multiple times to identify emerging themes and patterns [20,30,31]. Thematic analysis facilitated the identification of both explicit and implicit meanings in the data, offering a comprehensive understanding of the factors influencing children’s OH behaviors among Brazilian immigrant parents [31]. Data saturation was assessed throughout the analytic process and was considered to be reached when no new themes or meaningful insights emerged, which occurred after approximately 40 interviews [25,30,31].

Consistency and coding validity were maintained through regular team discussions and cross-checking of codes [25]. Because coding was primarily conducted by one researcher (D.L.N.) and reviewed by a second (A.C.L.), inter-rater reliability statistics such as Cohen’s Kappa could not be calculated. However, regular discussions and consensus-building throughout the coding process helped ensure the trustworthiness and consistency of the analysis.

Reflexivity was addressed by maintaining detailed field notes, where the researchers reflected on their potential biases and their impact on data collection and analysis [25]. The research team conducted regular debriefing sessions to discuss and mitigate biases, ensuring that the findings accurately represented the participants’ perspectives [20,25,30,31]. Cross-checking and team discussions validated the findings, while the final themes, written in English, were reviewed by a third research team member (M.L.G.) to ensure they accurately represented participants’ views and provided a comprehensive understanding of the factors influencing OH behaviors in Brazilian immigrant parents [20,30,31].

Finally, descriptive statistics (means, standard deviations, frequencies, and percentages) were used to summarize the sociodemographic characteristic data. The analyses were conducted using SAS 9.4 [33].

### 2.5. Ethical Considerations

Ethical approval was granted by the University of Massachusetts Boston Internal Review Board (IRB protocol #3541, approved 26 June 2023). All participants provided informed verbal consent, and confidentiality was ensured by anonymizing identifying information. Data were securely stored and accessible only to the research team. Participants were reminded of their right to withdraw at any time without consequences.

## 3. Results

### 3.1. Sociodemographics

Table 1 shows that the sample consisted of 48 Brazilian immigrant parents (29 mothers and 19 fathers), with a mean age of 36.5 years (SD = 6.6). Fathers were older on average (39.1 years, SD = 6.8) than mothers (33.9 years, SD = 6.5). The majority identified as mixed race (58.3%), followed by White (31.3%) and Black (10.4%). Most participants (85.4%) were married or living with a partner.

All participants were born in Brazil, with 58.2% from Minas Gerais. Half (56.3%) had lived in the U.S. for less than five years. Brazilian Portuguese was the primary language spoken at home, and 97.9% had a SASH score below 2.99, indicating low acculturation. In terms of education, 20.8% of participants had less than a high school diploma, 47.9% had completed high school, and 31.3% reported educational attainment more than high school.

The majority of participants’ household income was between USD 45,000 and USD 65,000 (52.1%). Most parents (70.8%) had one child aged 2–5, with 29.2% having two. The majority had public healthcare (91.7%) and dental insurance (68.7%).

### 3.2. Themes

Interviews revealed insights into how parents perceive the relationship between diet and OH, their strategies to promote healthy eating and oral hygiene habits, and the challenges they encounter in helping their children maintain both behaviors. The analysis identified three key thematic categories: (1) parental perceptions of diet’s influence on OH, (2) the role of parenting practices in fostering healthy eating and good OH habits, and (3) barriers to implementing and maintaining healthy eating and OH practices for their children. These themes are discussed in detail below.

#### 3.2.1. Parental Perceptions of Diets’ Influence on Oral Health

Parents consistently emphasized the critical role of diet in maintaining good OH for their children. They viewed healthy eating—especially diets rich in fruits and vegetables—as essential to preventing dental issues. Many parents described a holistic view of health, in which proper oral hygiene and nutritious diets are interconnected and mutually reinforcing.

Three key subthemes emerged from the analysis, highlighting parents’ understanding of the relationship between diet and their children’s OH, as well as the steps they take to promote healthy habits. These subthemes are (1) parents’ perceptions of the importance of proper oral hygiene and healthy eating habits, (2) parents’ recognition of sugary foods and beverages as major contributors to poor OH, and (3) strategies used by parents to limit their children’s intake of sugary foods and beverages. These subthemes are discussed in detail below.

Subtheme 1: Parents’ Perceptions of the Importance of Proper Oral Hygiene and Healthy Eating Habits

Most participants linked good oral health (OH) to both proper oral hygiene and healthy eating habits. They emphasized that a diet rich in fruits and vegetables is essential for preventing poor OH. For example, one father explained, *“An adequate diet, appropriate for their age, is important for their oral health”* (Father—40 years old, married, 2 years living in U.S.). Many also highlighted the importance of balancing diet and oral hygiene, noting that good habits in one area often led to good OH. One mother remarked, “*What the child eats, like fruits and other healthy foods. Of course, if they eat healthier foods, they will automatically have better oral health”* (Mother—26 years old, married, 4 years living in U.S.), while another mother reflected, *“You take care of yourself—your health, oral hygiene, and diet—all together”* (Mother—39 years old, married, 2 years living in U.S.).

Subtheme 2: Parents Recognize Sugary Foods and Beverages as Contributing to Poor Oral Health

Parents discussed the significant impact of sugary foods and beverages—especially sweets, soda, and processed foods—on OH. They emphasized that excessive sugar consumption, combined with poor oral hygiene, leads to cavities and other dental problems. One father observed, *“Sugar itself, right, because it causes cavities and everything else, it’s literally an enemy of the teeth”* (Father—33 years old, married, 5 years living in U.S.). Similarly, a mother added, *“When a child starts eating poorly, eating too many sweets, it really harms their oral health”* (Mother—35 years old, married, 6 years living in U.S.), while another explained, “*It’s because they eat too many sweets, drink soda, and don’t brush their teeth correctly”* (Mother—33 years old, married, 2 years living in U.S.). A father reinforced this belief, saying, *“If you don’t have good oral hygiene and eat a lot of sweets, those things can lead to problems”* (Father—52 years old, married, 2 years living in U.S.).

Subtheme 3: Strategies Used to Limit Sugary Foods and Beverages

Some parents highlighted their strategies to limit sugar intake and mitigate its effects on their children’s oral health. As one father shared, “*We avoid letting them [children] eat too much sugar*” (Father—52 years old, married, 2 years living in U.S.). Another explained their approach of brushing immediately after consuming sweets: “*I tell him to avoid candies and sweets, and when he does eat them, we brush his teeth right after*” (Father—34 years old, living with a partner/cohabiting, 2 years living in U.S.).

#### 3.2.2. The Role of Parenting Practices in Fostering Healthy Eating and Positive Oral Health Habits

Parents emphasized their role in helping children develop healthy eating habits, especially by limiting sugary foods. They recognized the need to balance nutrition with oral hygiene to prevent OH issues. Monitoring diet and educating children on healthy eating and hygiene were key strategies mentioned by parents.

Four subthemes related to the ways parents engage with their children’s nutrition and OH practices: (1) parental monitoring and control of sugary food intake, (2) bottle-feeding and its impact on OH, (3) parental involvement in educating and modeling healthy habits, and (4) mothers as the primary caregivers responsible for children’s OH were identified in the analysis. These subthemes are discussed in detail below.

Subtheme 1: Parental Monitoring and Control of Sugary Food Intake

Parents reported actively monitoring their children’s consumption of sugary foods, often setting clear limits and boundaries. Many spoke about limiting sugary foods in favor of healthier alternatives. One father explained, *“We avoid giving him sweets. He can eat more healthy food, like fruit”* (Father—33 years old, married, 2 years living in U.S.). While most parents recognized the impact of sugary foods on OH, nearly all allowed their children to have sweets in moderation on special occasions, like weekends. One father said, *“Sometimes we end up giving them some kind of sweet, something like that, as a reward for something nice they did”* (Father—38 years old, married, 2 years living in U.S.). Other parents spoke of serving lower-sugar treats to their children. For example, one mother mentioned, *“[…] I bought some chocolate powder that is seventy percent lower in sugar, it has less sugar than the regular one. Once in a while I add a little bit to her milk…”* (Mother—30 years old, separated, 2 years living in U.S.).

Subtheme 2: Bottle-Feeding’s Impact on Oral Health

Several parents reflected on how their early childhood feeding practices, particularly bottle-feeding, may impact their children’s OH. Some described using bottles with added sugars or sweeteners, such as ‘Mucilon,’ before bed. While parents expressed concern about their children consuming too much sugar, they still mentioned adding sweeteners to bottles, especially when preparing meals or snacks. One mother explained, *“I don’t put sugar in their bottles, because I know too much sugar isn’t good for them, especially when they’re little. But when I give them mococa (cornmeal porridge), I add a little sugar—just enough to make it taste better so they’ll eat it. I try to be careful, but sometimes you want them to enjoy the food too”* (Mother—26 years old, married, 3 years living in U.S.). This practice, common in Brazil, reflects a cultural tradition of adding sweetness to certain foods and drinks, even in the face of concerns about sugar intake, highlighting the challenge of balancing cultural habits with health considerations.

Furthermore, although some parents recognized that nighttime bottle use, when not followed by tooth brushing, affects OH, others mentioned instances where their children used bottles without subsequent oral hygiene practices. One mother explained*, “She sleeps with the bottle, and I don’t always make her brush her teeth afterward”* (Mother—30 years old, married, 5 years living in U.S.). In contrast, parents who had received professional advice were more mindful of the need for brushing after bottle use. One mother noted, *“I give him the bottle first, then brush his teeth, and put him to bed, as the pediatrician advised”* (Mother—33 years old, living a partner/cohabit, 7 years living in U.S.), demonstrating a more conscientious approach to managing OH after bottle use.

Subtheme 3: Parental Engagement in Educating, Modeling, and Supervising Healthy Eating and Oral Health Habits

Parents recognized the importance of educating their children about the link between diet and OH. Many emphasized the need to teach both the benefits of brushing and the importance of a balanced diet. One mother shared, *“I do everything I can to get them to eat the healthiest food available and brush their teeth after eating”* (Mother—35 years old, married, 6 years living in U.S.). Parents also saw themselves as role models for their children’s habits. As one father explained, “*I believe it’s about setting an example for them”* (Father—29 years old, living with a partner/cohabiting, 2 years living in U.S.).

Additionally, parents viewed their supervision of their children’s eating and hygiene habits as being crucial for maintaining good OH. One mother stated, *“I check every day to make sure they’re brushing their teeth properly and eating healthy”* (Mother—26 years old, married, 7 years living in U.S.). Many parents also emphasized the importance of brushing after consuming sweets. One father said, “I tell him to avoid candies and sweets, and when he does eat them, we brush his teeth right after” (Father—34 years old, living with a partner/cohabit, 2 years living in U.S.). However, some parents noted it is challenging to balance supervision with daily life, which sometimes led to compromises. One father mentioned, *“He doesn’t eat sweets all the time, but sometimes when he doesn’t want to stay still while I’m doing something, I give him a little treat”* (Father—39 years old, married, 3 years living in U.S.).

Subtheme 4: Mothers as Primary Caregivers for Oral Health and Healthy Eating

Many parents, both mothers and fathers, recognized that mothers are typically the primary caregivers responsible for their children’s OH and diet. Fathers often reported being less involved due to work schedules or other commitments. One father shared, *“I spend a lot of time outside the house, so when I come home, my child is already asleep”* (Father—40 years old, married, 2 years living in U.S.). Another father remarked, *“My wife doesn’t allow much. She’s very strict about sweets”* (Father—46 years old, married, 8 years living in U.S.). While some fathers expressed involvement, many acknowledged that mothers played a dominant role in managing their children’s OH. As one father explained, *“My wife takes on that role. I don’t have as much time”* (Father—29 years old, living with a partner/cohabiting, 2 years living in U.S.).

#### 3.2.3. Barriers to Implementing and Maintaining Healthy Eating and Oral Health Practices

Parents identified a variety of barriers to maintaining healthy eating practices for their children, influenced by a combination of social, economic, and logistical factors. These barriers included social pressures, financial constraints, and the challenges posed by demanding work schedules. Additionally, parents expressed difficulties in managing their children’s diets, oral hygiene, and OH due to external influences such as social gatherings, time limitations, and care provided by others. Additionally, several mentioned that economic challenges hindered their ability to provide healthy food options for their children consistently.

Two key subthemes emerged from the analysis, illustrating the diverse obstacles parents face in promoting healthy eating and OH. These subthemes are (1) social settings and external caregivers making it challenging to monitor children’s diets and oral hygiene and (2) the impact of work schedules and economic barriers on healthy eating and OH habits. These subthemes are discussed in detail below.

Subtheme 1: Social Settings and External Caregivers Making It Challenging to Monitor Children’s Diets and Oral Hygiene

Social occasions, such as children’s parties and events, were frequently identified as significant barriers to managing children’s diets and OH. Parents expressed frustration over the difficulty of limiting their children’s sugary treats at schools when other children were eating similar foods. One father explained, *“When there’s something festive, like at school, and other children are eating, we also let him have some chocolate or something, but it’s very rare”* (Father—40 years old, married, 2 years living in U.S.). Similarly, social gatherings, like birthday parties or visiting friends’ houses, posed challenges in limiting sugary snacks. One mother shared, “*When they’re at a friend’s house or somewhere else, we don’t have control. I know they come home really excited, and I know they’ve eaten a lot of things like that”* (Mother—26 years old, married, 3 years living in U.S.). Parents felt unable to regulate their children’s food choices outside the home, leading to potential risks for poor OH.

The difficulty of monitoring children’s eating habits was particularly evident when they were in the care of others, such as relatives and daycare providers. Parents expressed concern that when children were outside the home, they lost control over their eating routines. One mother explained, *“As soon as they are away from us, and we don’t have control over what’s in front of them, we can’t prevent other people from giving them certain types of food”* (Mother—30 years old, separated, 2 years living in U.S.). This lack of supervision was especially concerning when children were attending daycare or spending time with extended family members. One father shared, *“I know that I’m doing my part, I’m taking care of them, but I don’t know when they are away, if someone else would do the same”* (Father—46 years old, married, 8 years living in U.S.), making it difficult to maintain consistent dietary habits.

Several parents highlighted that a lack of supervision outside the home could undermine their efforts to promote healthy eating and oral hygiene for their children. One father shared, *“Without supervision, they don’t brush their teeth, and the parents don’t regulate sweets”* (Father—35 years old, living with a partner/cohabiting, 3 years living in U.S.), while another noted, *“If parents allow their children to eat junk food without regulation, it harms their child’s oral health in the future”* (Father—29 years old, living with a partner/cohabiting, 2 years living in U.S.). These statements emphasized the importance of consistent supervision and guidance in fostering and maintaining healthy habits, reflecting the parents’ understanding of their critical role in supporting their children’s OH.

Subtheme 2: Impact of Work Schedules and Economic Barriers on Healthy Eating and Oral Health Habits

Parents frequently cited work schedules and daily life demands as significant barriers to maintaining healthy eating and oral hygiene practices consistently. The limited time and fatigue from work made it challenging for many to monitor their children’s eating habits. One father explained, *“The main factor for me is really the issue of time because on the days I work, I tend to be away from home for 13, 14 hours. So, sometimes I leave the house while he’s still sleeping, or sometimes I see him in the morning, but the time we have together is very short. When I get back home, he’s already asleep because I return after 10 PM”* (Father—38 years old, married, 22 years living in U.S.). This challenge was especially evident for parents working long hours, leaving them little time to supervise their children’s eating and oral hygiene routines.

Additionally, many parents reported that financial limitations made it difficult to consistently afford nutritious foods. One father explained, *“Financially, we have to do the best we can within the house here because it’s very limited”* (Father—35 years old, living with a partner/cohabiting, 3 years living in U.S.). Families with lower incomes, in particular, had more limited access to healthier food options, making it challenging to prioritize nutritious meals over cheaper, less healthy alternatives. Several participants expressed concern about the affordability of nutritious foods, as one mother shared, *“Having the means to provide good food, a good environment for buying food, to have access to healthy foods”* (Mother—35 years old, living with a partner/cohabiting, 3 years living in U.S.). These financial barriers compounded the difficulty in maintaining healthy eating habits.

## 4. Discussion

This study is the first, to our knowledge, to explore Brazilian immigrant parents’ perceptions and practices regarding diet, feeding, and OH for their preschool-aged children, providing valuable insights into how they understand the relationship between these factors. Both mothers and fathers recognized the connection, emphasizing the importance of limiting sugar intake and promoting good oral hygiene. The findings underscore the significant role parents play in shaping their children’s eating habits and OH practices.

Consistent with prior research, the majority of parents in this study demonstrated an understanding of the link between diet and OH, particularly the negative effects of sugary foods [34,35,36,37,38,39,40]. Many identified sugar—particularly from sweets and sugary drinks—as a major contributor to poor OH, which aligns with the existing literature emphasizing the role of sugar in dental problems [39,41]. However, while both mothers and fathers recognized this connection, mothers seemed to be more active than fathers in managing their children’s diet and oral hygiene routines. This finding mirrors previous studies, where mothers are often considered the primary caregivers responsible for children’s nutrition and hygiene [16,18,36].

Parents identified external factors, such as school settings, birthday parties, and social gatherings, as key challenges in controlling their children’s sugar intake and maintaining consistent oral hygiene practices. These social environments, where children were often exposed to sugary snacks and drinks, made it difficult for parents to regulate their children’s diet at home. One major barrier was the lack of supervision when children were outside the home, leading to frustration over limited control in these settings. This finding aligns with prior studies that highlight the influence of social and environmental factors on children’s dietary habits and OH practices [41,42]. These studies have shown that external settings, like schools and social events, can undermine efforts to maintain healthy behaviors at home. Our findings suggest the need for broader interventions targeting social environments—such as schools and community organizations—to create a more supportive atmosphere for promoting children’s health [41,42,43]. These challenges highlight the complexity of establishing and maintaining healthy habits for children. Time constraints, reliance on others for childcare, and external social influences made it difficult for parents to consistently implement healthy eating habits and oral hygiene routines. Nonetheless, parents in the current study were committed to setting boundaries, educating their children, and seeking a balanced approach to both diet and OH. 

In the current study, parenting practices were viewed as being crucial in promoting healthy eating and OH behaviors, and parents were committed to limiting sugary foods and educating their children on the importance of diet and OH. Several described strategies, such as restricting access to sugary snacks and encouraging immediate tooth brushing after consuming sugary foods [16,34]. These practices align with dental health recommendations, emphasizing dietary control and timely oral hygiene to reduce cavity risk. As seen in other studies, mothers appeared to be more active in managing diet and oral hygiene routines [16,17,18,36]. This further supports the notion that mothers are often the primary agents in promoting children’s health behaviors, particularly in early childhood.

Additionally, many parents adopted a proactive, educational approach by teaching their children about the long-term benefits of good OH and healthy eating, which aligns with public health recommendations that emphasize early literacy around OH [4,9,10,15]. However, the effectiveness of these efforts may largely depend on the parents’ ability to supervise and enforce these practices consistently. Many parents reported challenges in doing this, particularly in situations where they were not present. This highlights an important issue: while parents may have the knowledge and intent to guide their children, external factors often interfere with their ability to do so [13,16,34]. Prior studies have similarly found that while parents often have good intentions and knowledge, external factors such as school environments, social gatherings, and childcare arrangements can disrupt their ability to enforce healthy behaviors [16,18,41,43,44]. These findings further underscore the need for supporting parental efforts with broader community and public health interventions.

The influence of acculturation may also play a role in shaping parental practices. Nearly all participants (97.9%) in this study were classified as having low acculturation based on their SASH scores. This may reflect continued adherence to traditional Brazilian parenting and health practices, while also presenting challenges in navigating the U.S. healthcare and OH systems. Limited acculturation could influence parents’ ability to access oral health information and services in their preferred language or to fully engage with health-promoting resources available in their communities. Although our sample was predominantly low-acculturation, precluding meaningful comparisons between low- and high-acculturation groups, this context highlights the importance of culturally and linguistically tailored health promotion efforts for immigrant populations.

An important finding of this study was the range of barriers parents faced when promoting healthy eating and OH. Time constraints, lack of supervision outside the home, social influences (e.g., school activities), and economic limitations were frequently cited as obstacles to managing children’s diets and maintaining regular oral hygiene [1,2,3,4,6]. Many parents reported feeling overwhelmed by their busy schedules, which often left them unable to consistently supervise their children’s eating habits or ensure adherence to oral hygiene routines. These challenges have been well-documented in other studies on parental involvement in children’s health behaviors [1,2,3,4,6,12].

Economic challenges also emerged as a significant barrier. Parents noted that the high cost of healthy foods made it difficult for them to consistently provide balanced meals. This issue, particularly the affordability of fresh fruits and vegetables, is well-documented in the existing literature [36,39,43]. Immigrant families often face economic pressures that require both parents to work long hours, leaving little time or resources to prioritize healthy eating [4,12,15]. These financial limitations further complicate efforts to promote good OH, as the accessibility of healthy food is often limited. Addressing these economic barriers will require systemic changes to ensure that healthy food options are both affordable and available to all families.

### 4.1. Implications for Public Health and Future Research

The findings of this study have significant implications for public health strategies aimed at improving children’s OH. As diet plays a critical role in OH, public health interventions should prioritize raising parents’ awareness about the importance of diet, nutrition, and oral hygiene from an early age [36,39,43,44,45,46,47,48]. Public health campaigns should be holistic and target multiple settings such as home, daycare, and preschool settings, integrating dietary guidelines with oral care practices, with a particular emphasis on reducing sugar consumption [41,42,43].

Addressing the barriers to healthy eating identified in this study requires a community-based approach. Schools, daycare centers, and other community organizations are pivotal in shaping children’s dietary behaviors [41,42,43]. Public health initiatives should actively engage these institutions in promoting healthier food choices and oral hygiene practices [44]. Policies encouraging healthier preschool environments—such as eliminating sugary snacks from menus and incorporating OH education into curricula—can support parents’ efforts and improve children’s overall health outcomes [41,44,49,50].

Future research should explore how social contexts, particularly school and social settings, influence children’s eating behaviors. Understanding the role of daycare and preschool environments and socioeconomic factors is essential for designing targeted interventions [41]. Additionally, investigating how these factors interact may provide insights into effective strategies to mitigate the influence of external social pressures on preschool-age children’s dietary choices.

Given the economic barriers identified by parents, public health policy should prioritize addressing disparities in access to healthy foods. Policies aimed at improving food security and ensuring access to affordable, nutritious foods are critical in reducing the impact of financial constraints on children’s OH [49,50]. Programs that assist low-income families in accessing healthier food options could help improve children’s diets and overall health outcomes, including OH.

### 4.2. Limitations and Strengths

The findings of this study should be interpreted in light of several limitations. Selection bias may have influenced the results, as participants were recruited through purposive and snowball sampling, potentially overrepresenting individuals who are more engaged or who hold stronger views [20]. Recruitment through digital platforms may have unintentionally excluded parents with limited internet access or limited health literacy, reducing the diversity and representativeness of the sample. This may impact our understanding of barriers to health-promoting behaviors, particularly among more vulnerable subgroups. Additionally, the context-specific focus on one immigrant community from one state limits generalizability to other populations. Social desirability bias may have affected how participants reported behaviors and attitudes, and while rigorous steps were taken to ensure reliability, thematic analysis remains subject to researcher interpretation [20].

Although the study included mothers and fathers, participants were primarily women. This gender imbalance may reflect caregiving norms within the community and could have influenced the findings, with mothers’ perspectives being more prominent than fathers’ in the analysis. Although thematic saturation was achieved overall, the small number of fathers participating in the study limited the ability to examine differences between mothers’ and fathers’ perspectives. 

Despite these limitations, this study has several notable strengths. The use of semi-structured interviews enabled a rich, in-depth exploration of participants’ perspectives, revealing cultural nuances and contextual influences on parenting practices. Importantly, the study included fathers—a group often underrepresented in research on parenting and preschool-aged children—providing a more comprehensive view of family dynamics and caregiving roles. By focusing specifically on Brazilian immigrants, the study also addresses a critical gap in the literature and contributes culturally relevant insights to inform future interventions and policies.

## 5. Conclusions

In conclusion, this study highlights the significant role of parents in shaping their children’s diet and OH behaviors. It also underscores their many challenges in balancing nutrition and oral hygiene in Brazilian families who have immigrated to the U.S. While parents are generally aware of the importance of limiting sugar and promoting good oral care, external factors—such as time constraints, social settings, and economic limitations—often hinder their efforts. Addressing these barriers through community-based interventions, education, and public policy could support parents in fostering healthier eating and oral hygiene habits in their children, leading to improved OH outcomes.

## Figures and Tables

**Table 1:** The sociodemographic and cultural characteristics of the sample (*n* = 48).

Variables	Total *N* (%)	Fathers *n* (%)	Mothers *n* (%)
**Age (Mean, SD)**	36.5 (6.6)	39.1 (6.8)	33.9 (6.5)
**Race**			
White	15 (31.3)	6 (31.6)	9 (31.0)
Black	5 (10.4)	1 (5.3)	4 (13.8)
Mixed race (Pardo/mestizo)	28 (58.3)	12 (63.1)	16 (55.2)
**Marital status**			
Married/living with partner	41 (85.4)	19 (100)	22 (75.9)
Divorced/separated	3 (6.3)	0	3 (10.3)
Single	4 (8.3)	0	4 (13.8)
**Educational attainment**			
Less than high school diploma	10 (20.8)	6 (31.6)	4 (13.8)
High school graduate	23 (47.9)	7 (36.8)	16 (55.2)
More than high school	15 (31.3)	6 (31.6)	9 (31.0)
**Household income**			
<USD 45,000/year	14 (29.1)	6 (31.6)	8 (27.6)
≥USD 45,000/year–<USD 65,000	25 (52.1)	11 (57.9)	14 (48.3)
≥USD 65,000/year	9 (18.8)	2 (10.5)	7 (24.1)
**Number of children between 2 and 5 years**			
1	34 (70.8)	9 (47.4)	25 (86.2)
2	14 (29.2)	10 (52.6)	4 (13.8)
**Born in Brazil**			
Yes	48 (100)	19 (100)	29 (100)
**States of origin**			
Minas Gerais	28 (58.2)	8 (42.1)	20 (69.0)
São Paulo	4 (8.3)	2 (10.5)	2 (6.9)
Espírito Santo	5 (10.5)	2 (10.5)	3 (10.2)
Paraná	2 (4.2)	0	2 (6.9)
Amazonas	1 (2.1)	0	1 (3.5)
Rio Grande do Norte	1 (2.1)	0	1 (3.5)
Bahia	4 (8.3)	4 (21.0)	0
Rondônia	1 (2.1)	1 (5.3)	0
Alagoas	1 (2.1)	1 (5.3)	0
Rio de Janeiro	1 (2.1)	1 (5.3)	0
**Years of Residence in the U.S.**			
<5 years	27 (56.3)	13 (68.5)	14 (48.3)
≥5 years–<10 years	16 (33.3)	4 (21.0)	12 (41.4)
≥10 years	5 (10.4)	2 (10.5)	3 (10.3)
**Primary language spoken at home**			
Brazilian Portuguese	48 (100)	19 (100)	29 (100)
**SASH ^1^**			
<2.99	47 (97.9)	19 (100)	28 (96.6)
≥2.99	1 (2.1)	0	1 (3.4)
**Healthcare insurance**			
Public (MassHealth)	44 (91.7)	17 (89.5)	27 (93.1)
Private	4 (8.3)	2 (10.5)	2 (6.9)
**Dental Care Insurance**			
Yes (Public, MassHealth)	33 (68.7)	17 (89.5)	16 (55.2)
No	15 (31.3)	2 (10.5)	13 (44.8)

^1^ Short Acculturation Scale for Hispanics (SASH).

## Data Availability

The data underlying this study are not publicly available due to confidentiality agreements with participants. However, reasonable requests for data may be made to the corresponding author, and these will be considered on a case-by-case basis, in compliance with ethical and legal restrictions.

## Abbreviations

The following abbreviations are used in this manuscript:

MA	Massachusetts
OH	Oral Health
SASH	Short Acculturation Scale for Hispanics
SEM	Social Ecological Model
U.S.	United States
